# The role of humor in the psychosomatic health of older adults

**DOI:** 10.1192/j.eurpsy.2025.1728

**Published:** 2025-08-26

**Authors:** B. Eleni, E. Dragioti, N. Zagorianakou, A. Nakou, C. Tsironis, S. Mantzoukas, M. Gouva

**Affiliations:** 1 Department of Nursing, School of Health Sciences, Research Laboratory Psychology of Patients, Families & Health Professionals; 2 Department of Nursing, School of Health Sciences, Research Laboratory of Integrated Health, Care and Well-being, Ioannina, Greece

## Abstract

**Introduction:**

Humor is widely recognized for its potential to improve psychosomatic health, enhancing both physical and mental well-being.

**Objectives:**

To investigate the correlation between humor styles and psychosomatic health in older adults.

**Methods:**

A cross-sectional study was conducted with 83 older adults (41 females, 42 males), aged 65 to 94 years (mean age: 74.1, SD = 8.1). Participants completed a sociodemographic questionnaire, the 32-item Humor Styles Questionnaire, and the 36-Item Short Form Survey (SF-36). Linear regression analysis was used to examine the relationship between humor styles and psychosomatic health outcomes.

**Results:**

Self-Enhancing Humor. For Aggressive Humor, the mean was 22.7 (SD = 7.95), and for Self-Defeating Humor, 26.3 (SD = 8.70). The mean scores for Physical Health and Mental Health were 277 (SD = 84.5) and 272 (SD = 70.6), respectively. Self-Enhancing Humor had a significant positive effect on Mental Health (B = 3.458, SE = 0.893, p < 0.001), RF (B = 0.7659, SE = 0.375, p = 0.044), GH (B = 0.7113, SE = 0.234, p = 0.003), MH (B = 0.9711, SE = 0.228, p < 0.001), and SF (B = 0.7165, SE = 0.329, p = 0.033). Additionally, Self-Defeating Humor showed a significant negative effect on RE (B = -1.093, SE = 0.474, p = 0.024).

**Conclusions:**

The findings suggest that positive humor styles, particularly Self-Enhancing Humor, are strongly associated with better psychosomatic health in older adults. Incorporating humor-based interventions could be a valuable approach to enhancing psychosomatic health in this population.

**Disclosure of Interest:**

None Declared

